# Titanocenes as Photoredox Catalysts Using Green‐Light Irradiation

**DOI:** 10.1002/anie.202001508

**Published:** 2020-04-17

**Authors:** Zhenhua Zhang, Tobias Hilche, Daniel Slak, Niels R. Rietdijk, Ugochinyere N. Oloyede, Robert A. Flowers, Andreas Gansäuer

**Affiliations:** ^1^ Kekulé-Institut für Organische Chemie und Biochemie Universität Bonn Gerhard Domagk-Straße 1 53121 Bonn Germany; ^2^ Department of Chemistry Lehigh University Bethlehem PA 18015 USA

**Keywords:** catalysis, epoxides, light, radicals, titanocenes

## Abstract

Irradiation of Cp_2_TiCl_2_ with green light leads to electronically excited [Cp_2_TiCl_2_]*. This complex constitutes an efficient photoredox catalyst for the reduction of epoxides and for 5‐*exo* cyclizations of suitably unsaturated epoxides. To the best of our knowledge, our system is the first example of a molecular titanium photoredox catalyst.

## Graphical abstract

**Green light**! For the first time, a titanium complex, Cp_2_TiCl_2_, is used in photoredox catalysis. Irradiation with green light enables an efficient catalytic radical chemistry with epoxides under very mild conditions and with reagent control.

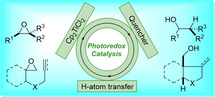

Due to the high abundance and low toxicity of the metal, the use of titanium catalysts is highly attractive for the development of sustainable reactions.^1^ Additionally, titanium complexes, like many other compounds of 3d metals, undergo facile one‐electron oxidation‐state changes. Therefore, they are attractive electron‐transfer catalysts in radical reactions. Given these advantages, it is surprising that in one of the most active areas of radical chemistry, photoredox catalysis,^2^ no titanium‐based catalysts have been introduced. Here, we describe the first examples of such reagents.2a

To this end, titanocene(IV) dihalides are especially attractive candidates. They are readily available in a large structural variety,^3^ they are colored and can absorb visible light, and, finally, the electron‐transfer chemistry of titanocene complexes has been successfully investigated in recent years.^4^ In agreement with previous studies,5a, 5b our TD‐DFT calculations (time‐dependent density functional theory; for details, see the Supporting Information)^6^ show that excitation results in an electron transfer from the highest occupied molecular orbital (HOMO) contributing to binding of titanium to the cyclopentadienyl ligands (Cp) to the mainly Ti‐centered lowest unoccupied molecular orbital (LUMO). This leads to a weakening of the bonding of the Cp ligands. Accordingly, the slow decomposition of photoexcited Cp_2_TiCl_2_ and Cp_2_TiBr_2_ occurs via loss of neutral C_5_H_5_ and not by loss of Cl or Br.5a The titanocenes exhibit an intense, long‐lived charge‐transfer phosphorescence at 77 K from the lowest ligand‐to‐metal charge‐transfer (LMCT) in the solid state (Cp_2_TiCl_2_: circa 800 μs).5b, 5c, 5d At 25 °C, the lifetimes are decreased even in the solid state.

We investigated the use of titanocene(IV) halides as photoredox catalysts in their most common application in electron‐transfer chemistry, the reductive opening of epoxides. To avoid the potential problem of short lifetimes of the photoexcited species, we examined the irradiation of Cp_2_TiX_2_ in the presence of a reductive quencher Q that also constitutes a potential ligand for titanium. Using this approach, it should be possible to generate the desired electron‐transfer catalyst Cp_2_TiX together with Q^.+^ without complete non‐radiative deactivation of the photoexcited [Cp_2_TiX_2_]*. Ideally, Q^.+^ can be used in the radical chemistry following the reductive epoxide opening (Scheme 1).

**Scheme 1:**
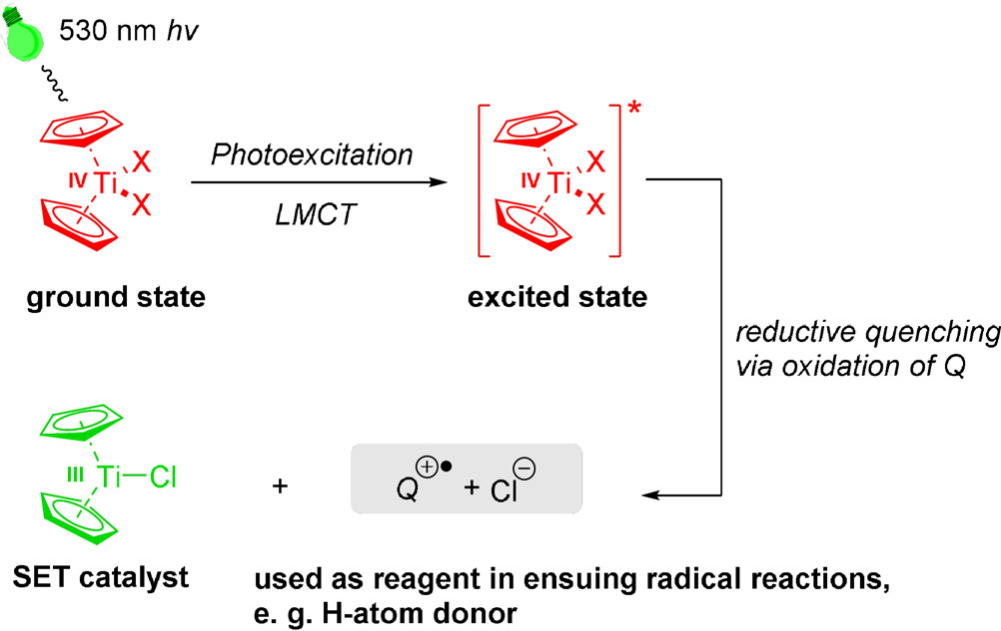
Concept of the use of photoexcited titanocenes in synthesis (LMCT=ligand to metal charge transfer; SET=single electron transfer).

Amines are attractive targets in this respect because they can be oxidized to the corresponding radical cations that are potential hydrogen‐atom donors.^7^ Our initial results for the reduction of **1** 8 in the presence of *N*,*N*‐diisopropyl ethylamine **3** (*i*Pr_2_NEt, Hünig's base, DIPEA) are summarized in Table 1.

**Table 1:** Optimization of the reduction of **1** with photoexcited Cp_2_TiCl_2_. 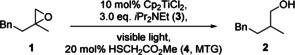

entry	visible light	MTG	yield [%]
1	blue LED	no	n.r.
2	blue LED	yes	52
3	green LED	yes	85
4	none	yes	n.r.
5	green LED	no	n.r.
6	green LED	yes	n.r.^[b]^
7	green LED	yes	n.r.^[c]^

[a] conditions: Cp_2_TiCl_2_ (10 mol %), **3** (3 equiv.), **4** (20 mol %), 0.1 m epoxide in tetrahydrofuran (THF), r.t., two 10 W LEDs. [b] no Cp_2_TiCl_2_. [c] no *i*Pr_2_NEt.

When only Cp_2_TiCl_2_ and **3** are irradiated with blue light in the presence of **1**, no **2** is formed (entry 1). This indicates that **3** ^.+^ is not a good‐enough hydrogen‐atom donor to reduce the radical formed through the opening of **1**. In the presence of the hydrogen‐atom‐transfer catalyst (HAT catalyst) methyl thioglycolate **4** (MTG),^9^ 52 % yield of **2** could be obtained (entry 2). Changing the source of light to a green light‐emitting diode (LED) lead to a noticeable improvement in the yield of **2** (85 %, entry 3). We propose that the lower wavelength of irradiation bypasses catalyst decomposition. The control experiments (entries 4–6) show that the presence of Cp_2_TiCl_2_, MTG, DIPEA, and light is mandatory for the conversion of **1** to **2**.

Next, we examined the influence of the amine and the counterion X of Cp_2_TiX_2_ on the performance of the reaction (Table 2).^10^ Of the other anions, only the use of Br results in a photoexcitation and a good yield of **1** (74 %) in the presence of **3**. Titanocenes with X=F, OMs, or O_2_CCF_3_ require light of higher energy for photoexcitation (see the Supporting Information for calculated spectra). With X=Cl, NEt_3_ leads to a noticeable lower yield than **3** and, curiously, in the presence of *i*PrNMe_2_, no product is formed at all.

**Table 2:** Influence of the amine and the anion X^−^ on the reduction of **1**. 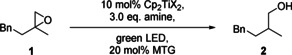

entry	X	amine	yield [%]
1	F	*i*Pr_2_NEt	n.r.
2	Br	*i*Pr_2_NEt	74
3	OMs	*i*Pr_2_NEt	n.r.
4	O_2_CCF_3_	*i*Pr_2_NEt	n.r.
5	Cl	NEt_3_	49^[b]^
6	Cl	*i*PrNMe_2_	n.r.

[a] conditions: Cp_2_TiX_2_ (10 mol %), amine (3 equiv.), **4** (20 mol %), 0.1 m epoxide in THF, r.t., two 10 W green LEDs. [b] by ^1^H NMR spectroscopy against an internal standard (1,3,5‐trimethoxylbenzene).

In Figure 1, the absorption and emission spectra of a solution of 0.01 mm Cp_2_TiCl_2_ in tetrahydrofuran (THF) at −78 °C are shown. We attribute the emission to the transition from the first triplet state of photoexcited Cp_2_TiCl_2_ to the ground state.5a, 5b At room temperature, the emission is not observed, suggesting that the lifetime of the transition is transient. The success of the reductive epoxide opening demonstrates that the triplet state does have a lifetime in THF at room temperature that is long enough for our purposes. The bimolecular quenching constant for photoexcited Cp_2_TiCl_2_ and DIPEA was determined by Stern–Volmer analysis and determined to be 1×10^4^ at −78 °C. This data clearly demonstrates the reduction by DIPEA (see the Supporting Information for details). Interestingly, other amines including triphenylamine are not effective quenchers. The proposed mechanism (Figure 2) features the formation of Cp_2_TiCl from photoexcited Cp_2_TiCl_2_ by reductive quenching with DIPEA and epoxide opening to yield the β‐titanoxy radical **A** that is reduced via the HAT cycle involving MTG (**4**). Because of the absence of vibrations due to an OH group in the IR spectrum of the reaction solution, **2** is not formed in the reaction. We propose the hemiaminal (**B**) as the initial product.

**Figure 1:**
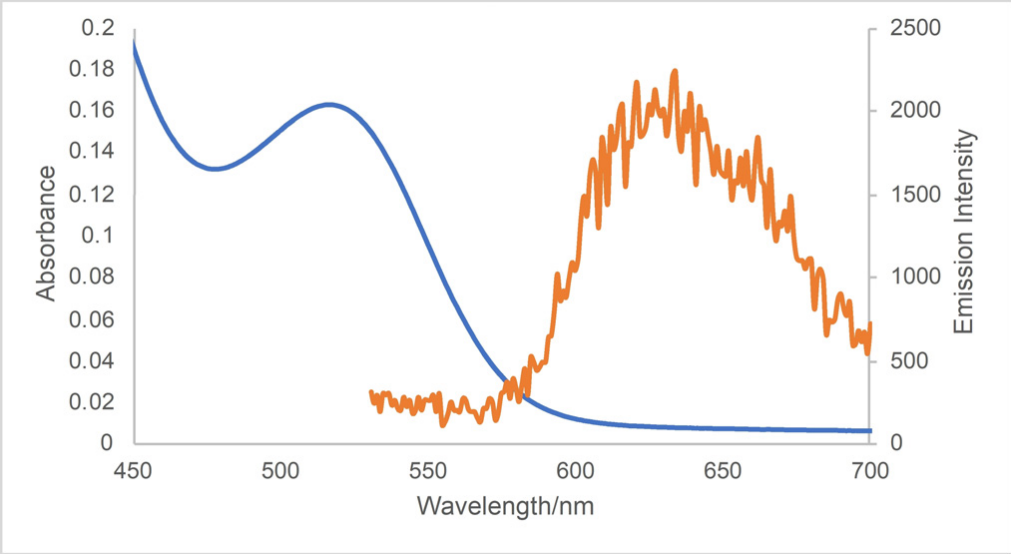
Absorption (blue line) and emission spectra (orange line) of Cp_2_TiCl_2_ in THF at −78 °C.

**Figure 2:**
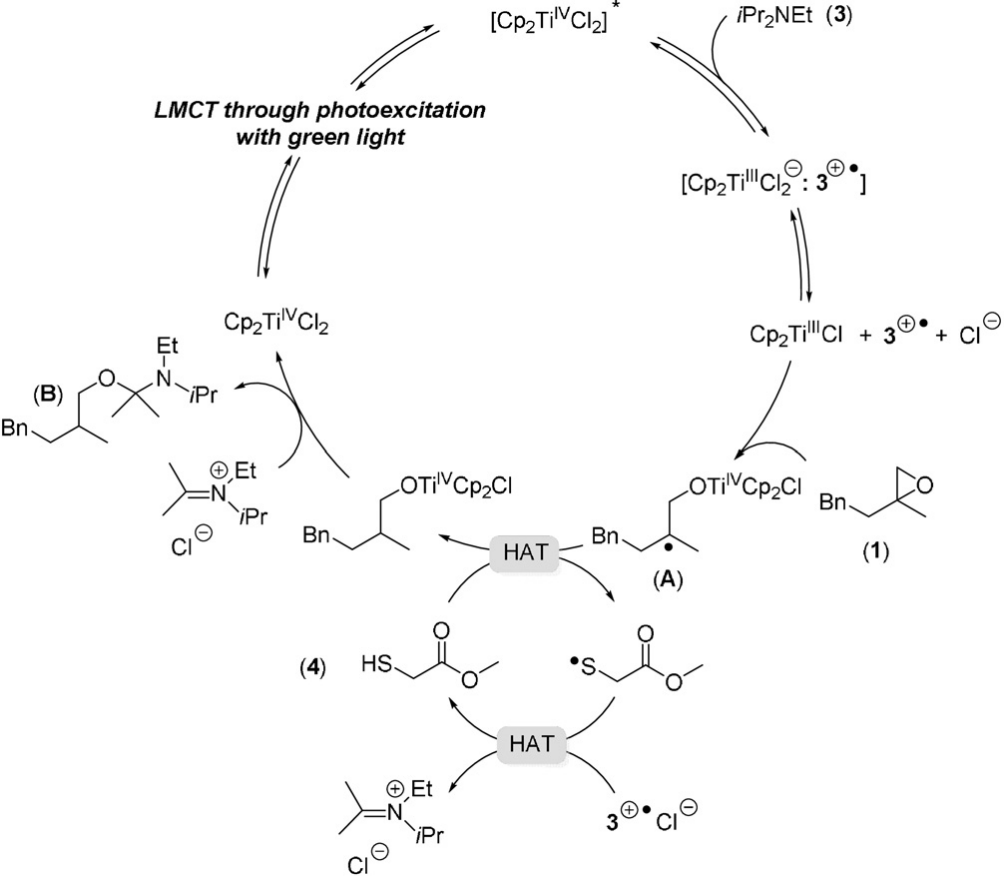
Proposed mechanism for epoxide reduction in the presence of photoexcited Cp_2_TiCl_2_.

We investigated the substrate scope of the epoxide reduction next (Table 3). Monosubstituted, 1,2‐/1,1‐disubstituted, and trisubstituted epoxides are suitable substrates for our reaction. The diastereoselectivities of the radical reduction (entries 3–5) are typical for radical reactions and rule out different mechanisms of epoxide opening. Gratifyingly, monosubstituted epoxides are opened with a much higher regioselectivity than in Mn‐ or Zn‐based systems.4l

**Table 3:** Scope of the epoxide reduction by photoexcited Cp_2_TiCl_2_. 
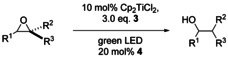

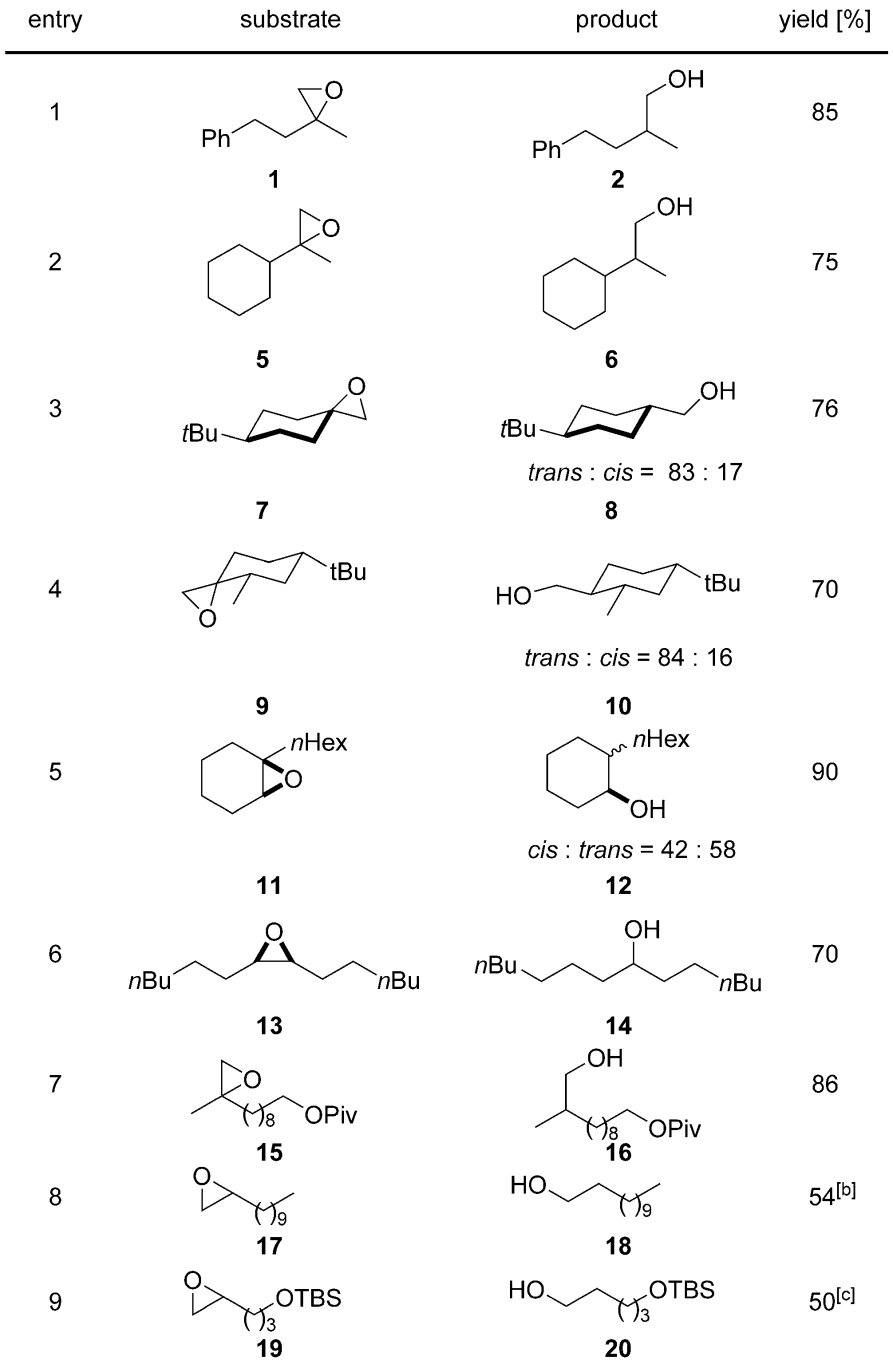

[a] conditions: Cp_2_TiCl_2_ (10 mol %), **3** (3 equiv.), **4** (20 mol %), 0.1 m epoxide in THF, r.t., two 10 W green LEDs. [b] **17** (0.05 m in THF), 50 °C, 94:6 mixture of 1‐and 2‐dodecanol. [c] **19** (0.05 m in THF), 50 °C, 96:4 mixture of 1,5‐ and 1,4‐diol. TBS=*t*BuMe_2_Si.

Arguably, the synthetically most important applications of radical chemistry are cyclizations. We investigated examples of the most prominent reaction in this respect, the 5‐*exo* cyclization.^11^, ^12^ Initial experiments were carried out under the conditions of reductive epoxide opening. However, with **21**, the desired product was obtained in a messy reaction with low yield (Table 4). We attribute the failure of the conditions to a competition of the reduction of the thiyl radical by **3** ^.+^ (the HAT cycle of Figure 2) with an addition of the thiyl radical to the double bond of **21**. This process is well known as key‐step in thiol–ene reactions and will result in a diminished efficiency of radical reduction.^13^ The addition of PhSiH_3_ (2.5 equiv.) to the reaction mixture and a change of the HAT catalyst to *n*‐octyl thioglycolate **23** (OTG) resolved this issue (Scheme 2) and lead to **22** in 80 % yield.^14^ A yield of 75 % was obtained with **4** as the HAT catalyst. The use of 0.5 equiv. of PhSiH_3_ also gave **22**, but in lower yields.

**Scheme 2:**
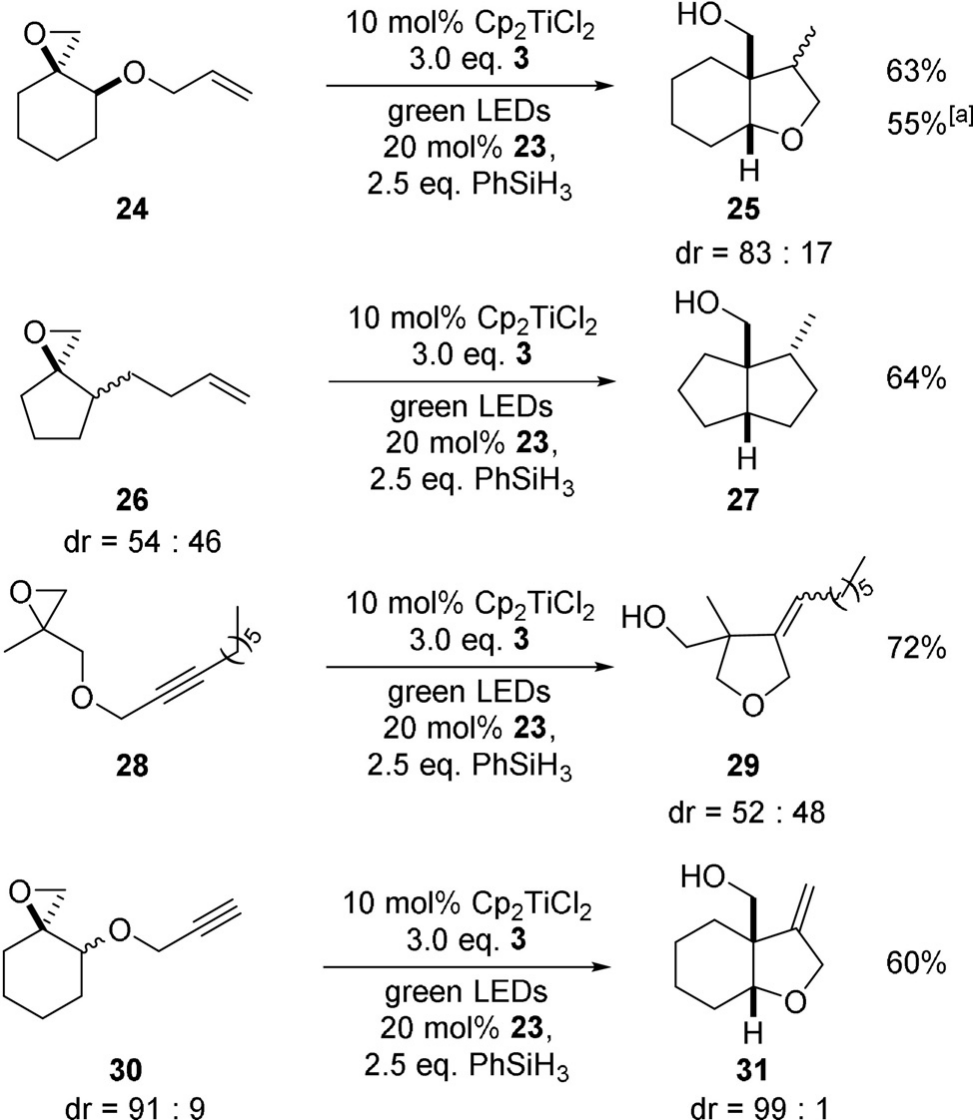
Examples of 5‐*exo* cyclizations with photoexcited Cp_2_TiCl_2_. [a] 1 equiv. PhSiH_3_.

**Table 4:** Optimization of the cyclization of **21** with photoexcited Cp_2_TiCl_2_. 
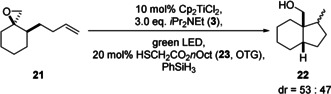

entry	PhSiH_3_ [equiv.]	yield [%]
1	–	40^[b,c]^
2	0.5	75
3	2.5	80
4	2.5	68^[c]^

[a] conditions: Cp_2_TiCl_2_ (10 mol %), **3** (3 equiv.), **23** (20 mol %), PhSiH_3_ (0.5–2.5 equiv.), 0.05 m epoxide in THF, r.t., two 10 W green LEDs. [b] by 1H NMR spectroscopy against an internal standard (1,3,5‐trimethoxybenzene). [c] MTG (**4**) used.

The other examples show that tetrahydrofurans can be readily accessed with our method. Alkynes are suitable radical acceptors. Once again, the diastereoselectivity of the reactions is typical for radical reactions and identical to those performed with Cp_2_TiCl formed by the reduction of Cp_2_TiCl_2_ with Zn or Mn.11b, 12

In summary, we have shown that Cp_2_TiCl_2_ which is irradiated with green light constitutes an efficient photocatalyst for epoxide‐derived radical chemistry. A key aspect of our approach is the use of *i*Pr_2_NEt as an oxidative quencher for [Cp_2_TiCl_2_]*. The activation of *i*Pr_2_NEt^+.^ for HAT is crucial for closing the catalytic cycles. To the best of our knowledge, we have presented the first example of photoredox chemistry catalyzed by a molecular titanium complex.

## Conflict of interest

The authors declare no conflict of interest.

## Supporting information

As a service to our authors and readers, this journal provides supporting information supplied by the authors. Such materials are peer reviewed and may be re‐organized for online delivery, but are not copy‐edited or typeset. Technical support issues arising from supporting information (other than missing files) should be addressed to the authors.

Supplementary
