# Supplementary material for: Titanocenes as Photoredox Catalysts Using Green‐Light Irradiation
Source: Angew Chem Int Ed Engl. 2020 Apr 17;59(24):9355–9. doi: 10.1002/anie.202001508 (PMC7317808; doi:10.1002/anie.202001508)
Supplement: Supplementary file 1 — Supplementary [file ANIE-59-9355-s001.pdf]

Angewandte Chemie
Eine Zeitschrift der Gesellschaft Deutscher Chemiker

Supporting Information

Titanocenes as Photoredox Catalysts Using Green-Light Irradiation

Zhenhua Zhang, Tobias Hilche, Daniel Slak, Niels R. Rietdijk, Ugochinyere N. Oloyede, Robert A. Flowers II, and Andreas Gansäuer**

anie_202001508_sm_miscellaneous_information.pdf

Table of Contents

1. General Information 3

2. General Procedure for the Reduction of Epoxides (G1) 3

3. General Procedure for the Cyclization of Epoxides (G2) 4

4. Preparation of Tertiary Amines and Phosphate Buffer 4

5. Syntheses of Substrates and Catalysts 5

6. Synthesis of Products 5

7. Mechanistic Studies 11

   7.1. Detection of Product 2 through IR Experiment 11

   7.2. Detection of Product 2 through ReactIR Experiment 12

   7.3 Absorption-Emission spectra 13

   7.4. Luminescence Quenching Experiments and Stern-Volmer Plot 13

8. Computational Results 16

   8.1. General remarks, geometry optimizations and UV/vis spectra calculations 16

   8.2. UV/vis spectra and HOMO-LUMO transitions 16

   8.3. Discussion of Anion Effect 16

References 20

1. General Information

All reactions involving air or moisture sensitive compounds were carried out in oven-dried glassware under argon using standard Schlenk and vacuum line technique. All solvents were either dried and deoxygenated by distillation before use or purified inside an M-Braun MB-SPS-800 solvent purification system. All reactions were monitored by thin-layer chromatography (TLC) on Merck silica gel 60 F254 plates using UV light as visualizing agent (if applicable), and a solution of ammoniummolybdate tetrahydrate (25 g•L^{-1}) and $Ce(SO_4)_2$•$4H_2O$ (10 g•L^{-1}) in 10% aqueous H_2SO_4 followed by heating as developing agents. Products were purified by flash column chromatography on Merck silica gel 50 or Macherey-Nagel silica gel 60. ^1H NMR and ^{13}C NMR spectra were measured on Bruker AMX 300 MHz or Bruker 400 MHz or 500 MHz spectrometers. ^1H NMR chemical shift were given in ppm, and calibrated by using the residual undeuterated solvent as internal reference ($CHCl_3$ (7.26 ppm), d_5-benzene (7.16 ppm)). ^{13}C NMR chemical shift were recorded in ppm and the solvent peak was employed as internal reference ($CDCl_3$ (77.0 ppm), C_6D_6 (128.0 ppm)). IR spectra were measured on an ATR-IR-Spectrometer Nicolet TM 380 instrument as neat film. High-resolution mass spectra analysis data were obtained on a Thermoquest MAT 95 XL instrument. The light source is Eurolite LED IP FL-10 Outdoor LED-Spot Anzahl LEDs (10 W).

2. General Procedure for the Reduction of Epoxides (G1)

To a heat-dried Schlenk tube epoxide (0.5 mmol, 1 eq.), Cp_2TiCl_2 (12.5 mg, 0.05 mmol, 10.0 mol%), methyl thioglycolate (MTG, 8.0 μL, 0.10 mmol, 20 mol%), *N,N*-diisopropyl ethyl amine (DIPEA, 0.26 mL, 1.5 mmol, 3.0 eq.) and 5 mL tetrahydrofuran (THF) were added under argon atmosphere. The mixture was then stirred rapidly and irradiated with two 10 W green light emitting diods (LEDs) at room temperature for 48h. Then, 10 mL phosphate buffer was added to the mixture. The resulting mixture was sequentially extracted by diethyl ether, washed with water and brine and dried over Na_2SO_4. The resulting mixture was carefully concentrated under vacuum, affording crude product. This was purified by flash column chromatography on silica gel with ethyl acetate and cyclohexane as eluent.

3. General Procedure for the Cyclization of Epoxides (G2)

To a heat-dried Schlenk tube epoxide (0.5 mmol, 1 eq.), Cp_2TiCl_2 (12.5 mg, 0.05 mmol, 10.0 mol%), *n*-octyl thioglycolate (OTG, 21 μL, 0.10 mmol, 20 mol%), DIPEA (0.26 mL, 1.5 mmol, 3.0 eq.), $PhSiH_3$ (0.5-2.5 eq.) and 10 mL THF were added under argon atmosphere. The mixture was then stirred rapidly and irradiated with two 10 W green LEDs at room temperature for 48h. NaOH solution (2 M in water, 2 mL) was added dropwise over 5 min. The resulting mixture was diluted with water (10 mL) and extracted with diethyl ether (3 x 20 mL). The combined organic phases were washed with brine and dried over Na_2SO_4, and the solvent was removed under reduced pressure. The residue was purified by flash column chromatography on silica gel.

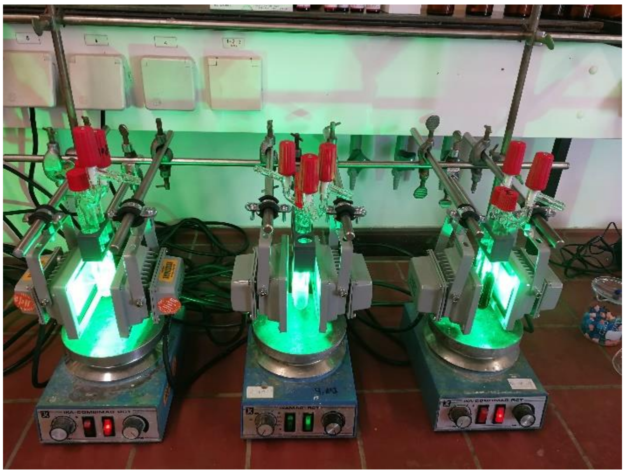

4. Preparation of Tertiary Amines and Phosphate Buffer

Calcium hydride was added to tertiary amines and the mixture was stirred for 2 h. After distillation under normal pressure and argon, the dry tertiary amines were stored with 4 Å molecular sieves (10 wt%) under argon.

Phosphate buffer (pH = 7): H_2O (400 mL), H_3PO_4 (18.4 g of a 85% solution in H_2O) and KH_2PO_4 (10.9 g).

5. Syntheses of Substrates and Catalysts

Cp_2TiCl_2, methyl glycolate, *n*-octyl glycolate and 2-decyloxirane were commercially available and directly used without further purification.

The following epoxides and photocatalyst were prepared according to the literature.

2-methyl-2-phenethyloxirane.[1]

2-cyclohexyl-2-methyloxirane.[2]

6-(*tert*-butyl)-1-oxaspiro[2.5]octane.[3]

6-(*tert*-butyl)-4-methyl-1-oxaspiro[2.5]octane.[4]

1-hexyl-7-oxabicyclo[4.1.0]heptane.[5]

2,3-dihexyloxirane.[6]

2,3-bis(ethoxymethyl)oxirane.[7]

2-(2-methyloxiran-2-yl)ethyl pivalate.[1]

tert-butyldimethyl(3-(oxiran-2-yl)propoxy)silane.[8]

4-(but-3-en-1-yl)-1-oxaspiro[2.5]octane.[9]

4-(allyloxy)-1-oxaspiro[2.5]octane.[9]

4-(but-3-en-1-yl)-1-oxaspiro[2.4]heptane.[9]

2-methyl-2-((non-2-yn-1-yloxy)methyl)oxirane.[9]

4-(but-3-yn-1-yl)-1-oxaspiro[2.5]octane.[9]

4-(prop-2-yn-1-yloxy)-1-oxaspiro[2.5]octane.[9]

6. Synthesis of Products

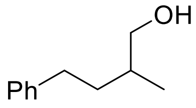

2-methyl-4-phenylbutan-1-ol (2)

According to GP 1, **1** (81 mg, 0.50 mmol, 1.0 eq.), Cp_2TiCl_2 (12.5 mg, 0.05 mmol, 10.0 mol%), MTG (8.0 µL, 0.10 mmol, 20 mol%) and DIPEA (0.26 mL, 1.5 mmol, 3.0 eq.) in THF (5 mL) were stirred at room temperature under green LED light for 48 h. Chromatography on silica yielded the product **2** (70 mg, 0.43 mmol, 85%).

The NMR data is in agreement with the literature.[10]

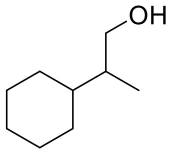

2-cyclohexylpropan-1-ol (6)

According to GP 1, **5** (70 mg, 0.50 mmol, 1.0 eq.), Cp_2TiCl_2 (12.5 mg, 0.05 mmol, 10.0 mol%), MTG (8.0 µL, 0.10 mmol, 20 mol%) and DIPEA (0.26 mL, 1.5 mmol, 3.0 eq.) in THF (5 mL) were stirred at room temperature under green LED light for 48 h. Chromatography on silica yielded the product **6** (53 mg, 0.38 mmol, 75%).

The NMR data is in agreement with the literature.[10]

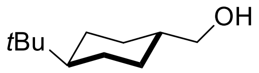

trans: cis= 83: 17

4-(tert-butyl)cyclohexyl)methanol (8)

According to GP 1, **7** (84 mg, 0.50 mmol, 1.0 eq.), Cp_2TiCl_2 (12.5 mg, 0.05 mmol, 10.0 mol%), MTG (8.0 µL, 0.10 mmol, 20 mol%) and DIPEA (0.26 mL, 1.5 mmol, 3.0 eq.) in THF (5 mL) were stirred at room temperature under green LED light for 48 h. Chromatography on silica yielded the product **8** (54 mg, 0.38 mmol, 76%).

The NMR data is in agreement with the literature.[3]

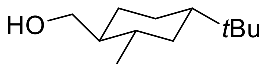

d.r. =84:16

4-(tert-butyl)-2-methylcyclohexyl)methanol (10)

According to GP 1, **9** (91 mg, 0.50 mmol, 1.0 eq.), Cp_2TiCl_2 (12.5 mg, 0.05 mmol, 10.0 mol%), MTG (8.0 µL, 0.10 mmol, 20 mol%) and DIPEA (0.26 mL, 1.5 mmol, 3.0 eq.) in THF (5 mL) were stirred at room temperature under green LED light for 48 h. Chromatography on silica yielded the product **10** (64 mg, 0.35 mmol, 70%).

The NMR data is in agreement with the literature.[6]

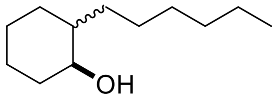

cis: trans=42 :58

2-hexylcyclohexan-1-ol (12)

According to GP 1, **11** (91 mg, 0.50 mmol, 1.0 eq.), Cp_2TiCl_2 (12.5 mg, 0.05 mmol, 10.0 mol%), MTG (8.0 µL, 0.10 mmol, 20 mol%) and DIPEA (0.26 mL, 1.5 mmol, 3.0

eq.) in THF (5 mL) were stirred at room temperature under green LED light for 48 h. Chromatography on silica yielded the product **12** (83 mg, 0.45 mmol, 91%).
The NMR data is in agreement with the literature.[5]

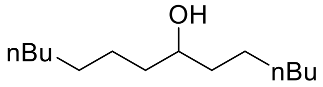

tetradecan-7-ol (14)
According to GP 1, **13** (106 mg, 0.50 mmol, 1.0 eq.), Cp_2TiCl_2 (12.5 mg, 0.05 mmol, 10.0 mol%), MTG (8.0 µL, 0.10 mmol, 20 mol%) and DIPEA (0.26 mL, 1.5 mmol, 3.0 eq.) in THF (5 mL) were stirred at room temperature under green LED light for 48 h. Chromatography on silica yielded the product **14** (75 mg, 0.35 mmol, 70%).
The NMR data is in agreement with the literature.[6]

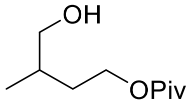

4-hydroxy-3-methylbutyl pivalate (16)
According to GP 1, **15** (93 mg, 0.50 mmol, 1.0 eq.), Cp_2TiCl_2 (12.5 mg, 0.05 mmol, 10.0 mol%), MTG (8.0 µL, 0.10 mmol, 20 mol%) and DIPEA (0.26 mL, 1.5 mmol, 3.0 eq.) in THF (5 mL) were stirred at room temperature under green LED light for 48 h. Chromatography on silica yielded the product **16** (81 mg, 0.43mmol, 86%).
The NMR data is in agreement with the literature.[1]

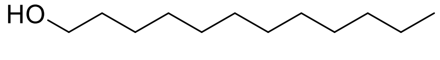

dodecan-1-ol

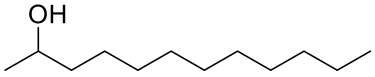

dodecan-2-ol

dodecan-1-ol (18)
According to GP 1, **17** (92 mg, 0.50 mmol, 1.0 eq.), Cp_2TiCl_2 (12.5 mg, 0.05 mmol, 10.0 mol%), MTG (8.0 µL, 0.10 mmol, 20 mol%) and DIPEA (0.26 mL, 1.5 mmol, 3.0 eq.) in THF (5 mL) were stirred at 50°C (oil bath) under green LED light for 48 h. Chromatography on silica yielded a 94 : 6 mixture of dodecan-1-ol **18** and dodecan-2-ol (50 mg, 0.27mmol, 54%).
The NMR data is in agreement with the literature.[11]

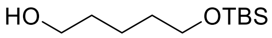

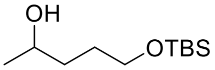

5-((*tert*-butyldimethylsilyl)oxy)pentan-1-ol **5-((*tert*-butyldimethylsilyl)oxy)pentan-2-ol**

5-((*tert*-butyldimethylsilyl)oxy)pentan-1-ol (20)

According to GP 1, **19** (108 mg, 0.50 mmol, 1.0 eq.), Cp_2TiCl_2 (12.5 mg, 0.05 mmol, 10.0 mol%), MTG (8.0 µL, 0.10 mmol, 20 mol%) and DIPEA (0.26 mL, 1.5 mmol, 3.0 eq.) in THF (10 mL) were stirred at 50°C (oil bath) under green LED light for 48 h. Chromatography on silica yielded a 96 : 4 mixture of 5-((*tert*-butyldimethylsilyl)oxy)pentan-1-ol **20** and 5-((*tert*-butyldimethylsilyl)oxy)- pentan-2-ol (55 mg, 0.25mmol, 50%).

The NMR data is in agreement with the literature.[12]

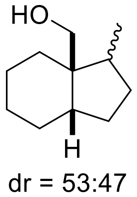

3-methyloctahydro-3aH-inden-3a-yl)methanol (22)

According to GP 2, **21** (83 mg, 0.50 mmol, 1.0 eq.), Cp_2TiCl_2 (12.5 mg, 0.05 mmol, 10.0 mol%), OTG (21 µL, 0.10 mmol, 20 mol%), DIPEA (0.26 mL, 1.5 mmol, 3.0 eq.) and $PhSiH_3$ (150 µL, 1.25 mmol, 2.50 eq.) in THF (10 mL) were stirred at room temperature under green LED light for 48 h. Chromatography on silica yielded the product **22** (67 mg, 0.40mmol, 80%).

The NMR data is in agreement with the literature.[13]

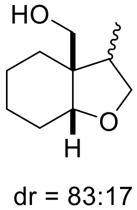

3-methylhexahydrobenzofuran-3a(4H)-yl)methanol (25)

According to GP 2, **24** (84 mg, 0.50 mmol, 1.0 eq.), Cp_2TiCl_2 (12.5 mg, 0.05 mmol, 10.0 mol%), OTG (21 µL, 0.10 mmol, 20 mol%), DIPEA (0.26 mL, 1.5 mmol, 3.0 eq.) and $PhSiH_3$ (150 µL, 1.25 mmol, 2.50 eq.) in THF (10 mL) were stirred at room temperature under green LED light for 48 h. Chromatography on silica yielded the product **25** (54 mg, 0.32mmol, 63%).

The NMR data is in agreement with the literature.[9]

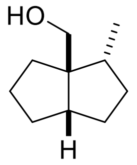

dr = 99:1

3-methylhexahydropentalen-3a(1H)-yl)methanol (27)

According to GP 2, **26** (76 mg, 0.50 mmol, 1.0 eq.), Cp_2TiCl_2 (12.5 mg, 0.05 mmol, 10.0 mol%), OTG (21 µL, 0.10 mmol, 20 mol%), DIPEA (0.26 mL, 1.5 mmol, 3.0 eq.) and $PhSiH_3$ (150 µL, 1.25 mmol, 2.50 eq.) in THF (10 mL) were stirred at room temperature under green LED light for 48 h. Chromatography on silica yielded the product **27** (49 mg, 0.32mmol, 64%).

The NMR data is in agreement with the literature.[13]

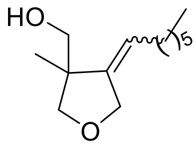

dr = 52:48

(3-methyl-4-propylidenetetrahydrofuran-3-yl)methanol (29)

According to GP 2, **28** (105 mg, 0.500 mmol, 1.00 eq.), Cp_2TiCl_2 (12.5 mg, 0.05 mmol, 10.0 mol%), OTG (21 µL, 0.10 mmol, 20 mol%), DIPEA (0.26 mL, 1.5 mmol, 3.0 eq.) and $PhSiH_3$ (150 µL, 1.25 mmol, 2.50 eq.) in THF (10 mL) were stirred at room temperature under green LED light for 48 h. Chromatography on silica yielded the product **29** (76 mg, 0.36mmol, 72%).

The NMR data is in agreement with the literature.[9]

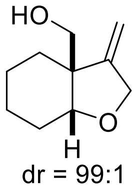

dr = 99:1

3-methylenehexahydrobenzofuran-3a(4H)-yl)methanol (31)

According to GP 2, **30** (83 mg, 0.500 mmol, 1.00 eq.), Cp_2TiCl_2 (12.5 mg, 0.05 mmol, 10.0 mol%), OTG (21 µL, 0.10 mmol, 20 mol%), DIPEA (0.26 mL, 1.5 mmol, 3.0 eq.) and $PhSiH_3$ (150 µL, 1.25 mmol, 2.50 eq.) in THF (10 mL) were stirred at room temperature under green LED light for 48 h. Chromatography on silica yielded the

product **31** (50 mg, 0.30mmol, 60%).

The NMR data is in agreement with the literature.[9]

7. Mechanistic Studies

7.1. Detection of Product 2 through IR Experiment

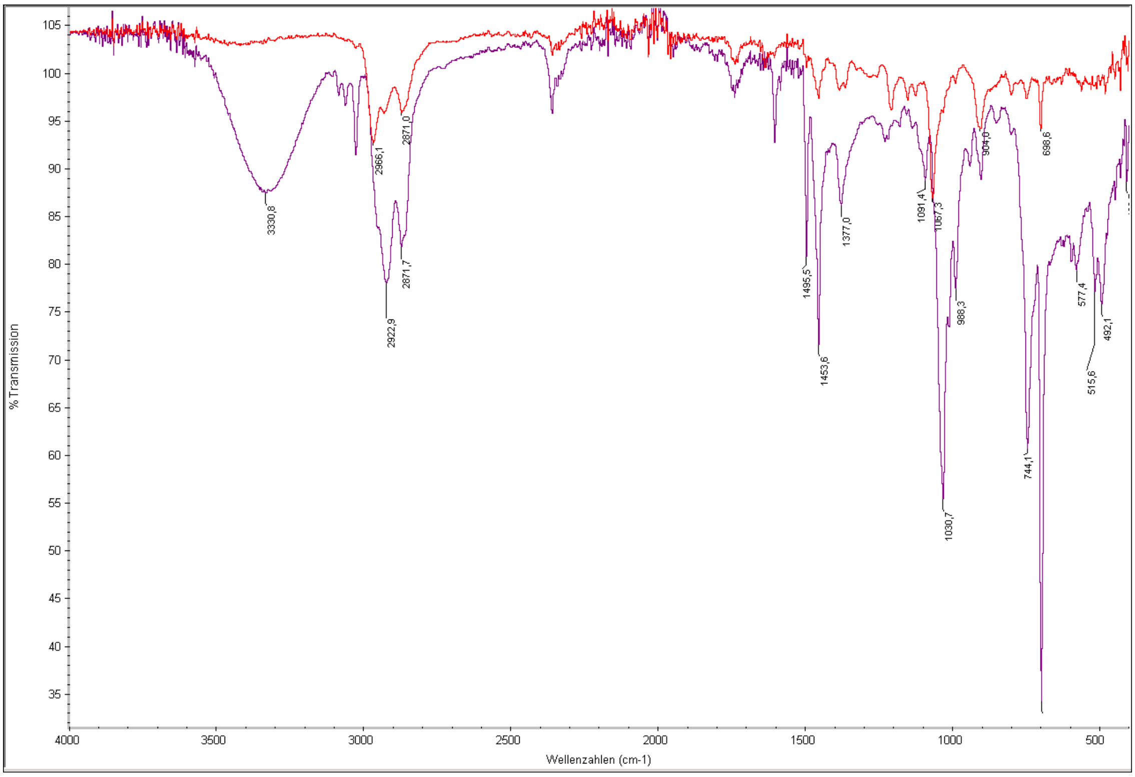

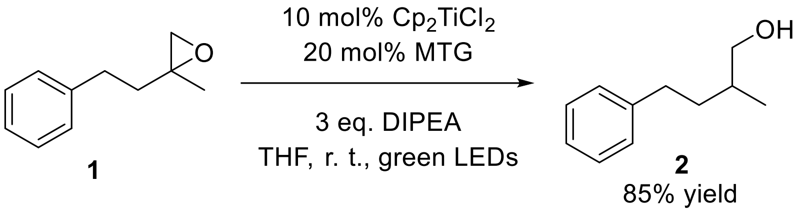

When this reaction was finished, the reaction mixture was directly measured with a Nicolet 380 FT-IR spectrometer to give a spectrum and the peak of OH group was not observed (red line). The position of this OH group was identified by the comparison with the spectrum of pure product (purple line, 3330 cm^{-1}).

7.2. Detection of Product 2 through ReactIR Experiment

The rate of conversion was monitored using a Mettler-Toledo's ReactIR 15 fitted with DiComp probe and running on the iCIR software 4.3 SP1. A solution of Cp_2TiCl_2 (0.05 mmol, 10.0 mol%, 12.5 mg), DIPEA (1.5 mmol, 3 eq., 0.26 ml), MTG (0.1 mmol, 20 mol%, 9.0 µL), and 2-methyl-2-phenethyloxirane (0.5 mmol, 1 eq., 81 mg) in dry degassed THF (5 ml) was prepared in an argon glove box. A two-necked round bottom

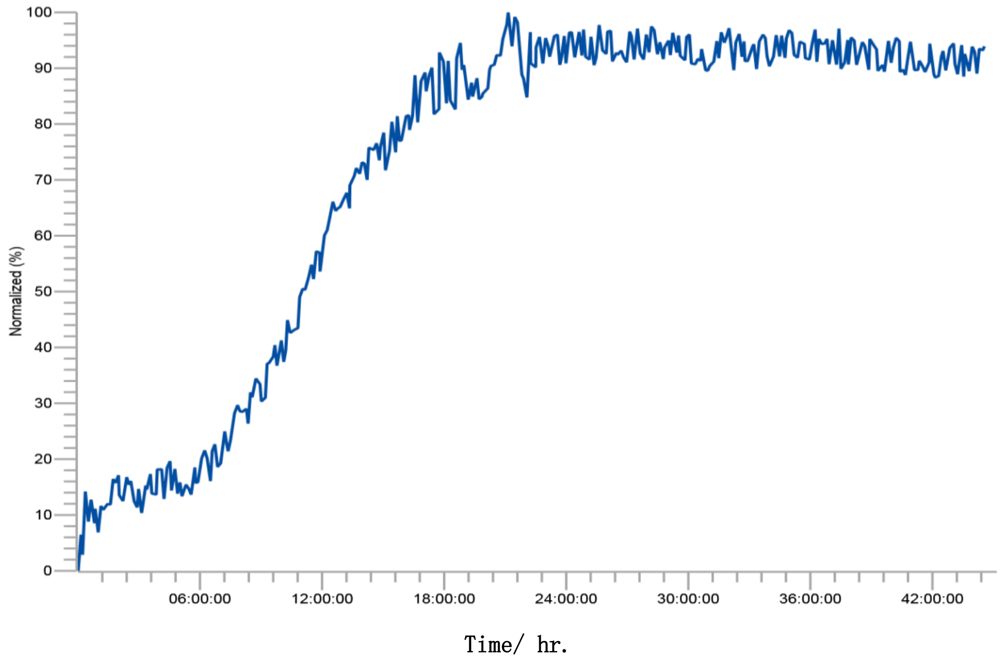

flask was fixed to the ReactIR probe, flushed with argon and scanned for background air (256 scans). The prepared solution was transferred to the argon filled round bottom flask and irradiated with green LEDs. The experiment was initiated on the software and data was collected every minute until completion of the reaction. Progress of the reaction was monitored at 1018 cm^{-1} which corresponds to the RH_2C-O_{str} of the product. Figure: Normalized conversion (%) of 2-methyl-2-phenethyloxirane to 2-methyl-4-phenylbutan-1-ol monitored at 1018 cm^{-1}.

7.3 Absorption-Emission spectra

The absorbance and the emission spectra were obtained using Cary 5000 UV-Vis spectrophotometer v3 and Fluorolog^{TM} Horiba Scientific Fluorometer v3.9 respectively.

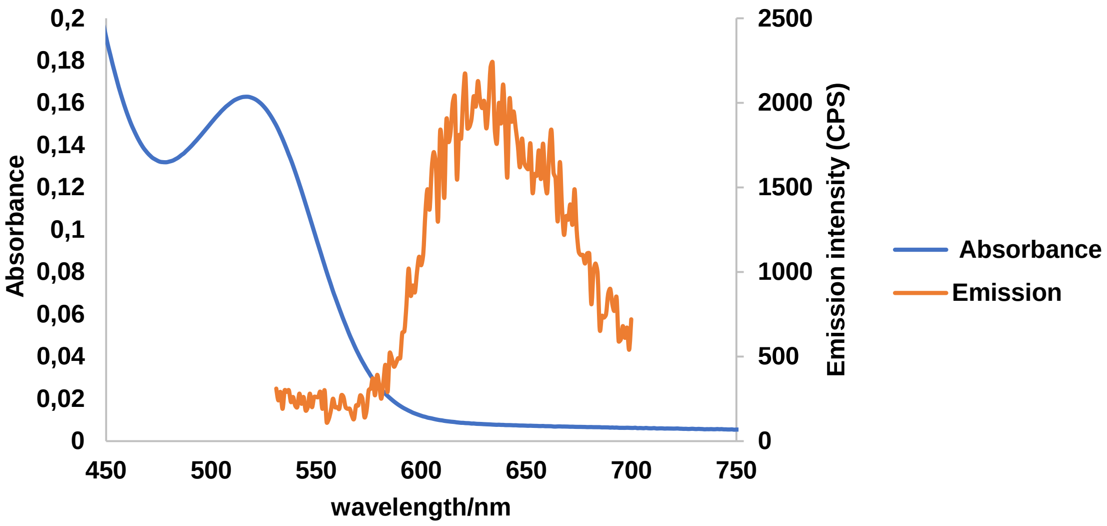

Figure: Overlay of the absorbance and emission of Cp_2TiCl_2 (0.01 mM) in THF.

7.4. Luminescence Quenching Experiments and Stern-Volmer Plot

Quenching analysis of the excited state titanocene, Cp_2TiCl_2*, was carried out on a Fluorolog^{TM} Horiba Scientific Fluorometer v3.9. The following parameters were applied for the experiment: bandpass= 3 nm, integration time= 0.1 s, grating density= 1200. Measurements were obtained at -78 ^{0}C.

Stern-Volmer plots were obtained using the Stern-Volmer kinetic equation;

$$I_0/I = K_{SV}\,[Quencher] + 1$$

Where I_0 is the luminescence intensity without the quencher, I is the luminescence intensity with the quencher and K_{SV} is the Stern-Volmer constant.

1 mM solution of Cp_2TiCl_2 (2.5 mg in 10 mL THF), 10 mM solution of diisopropylethylamine (DIPEA, 17 µL in 10 mL THF) and 10 mM solution of triphenylamine (TPA, 24.5 mg in 10 mL THF) were prepared. 50 µL aliquots of the 1 mM solution of Cp_2TiCl_2 was added to 10 µL, 25 µL, 50 µL, 100 µL and 150 µL aliquots of the 10 mM solution of Diisopropylethylamine or Triphenylamine respectively in a 5 mL standard flask and made up to the mark with THF. The prepared solutions were analyzed on the fluorometer.

Figure: Quenching and Stern-Volmer analysis of Cp_2TiCl_2 and DIPEA (quencher)

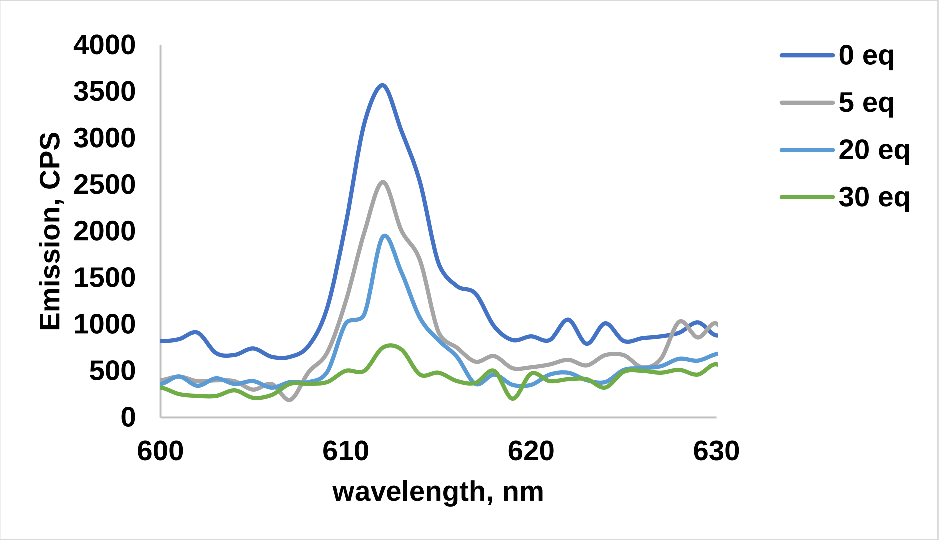

a.

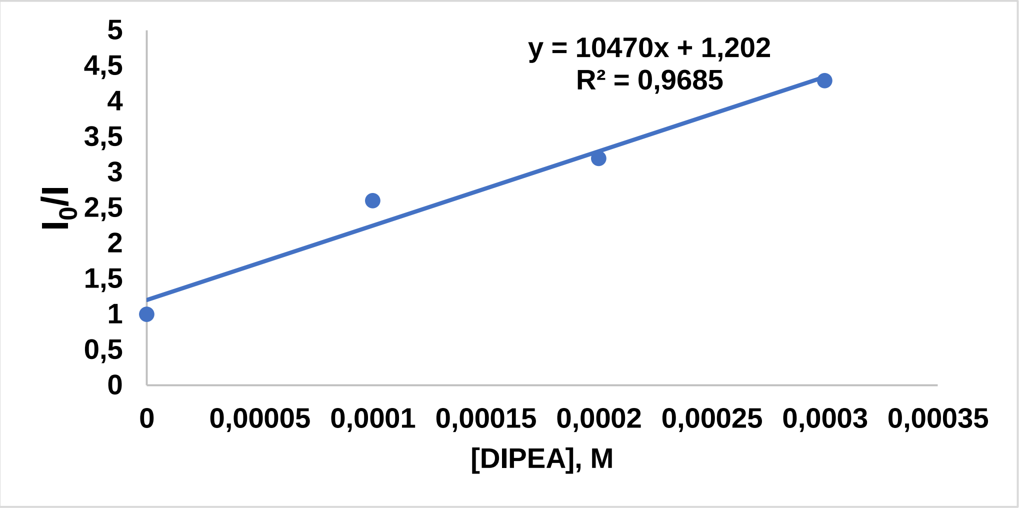

b.

Figure: Quenching and Stern-Volmer analysis of Cp_2TiCl_2 and TPA (quencher)

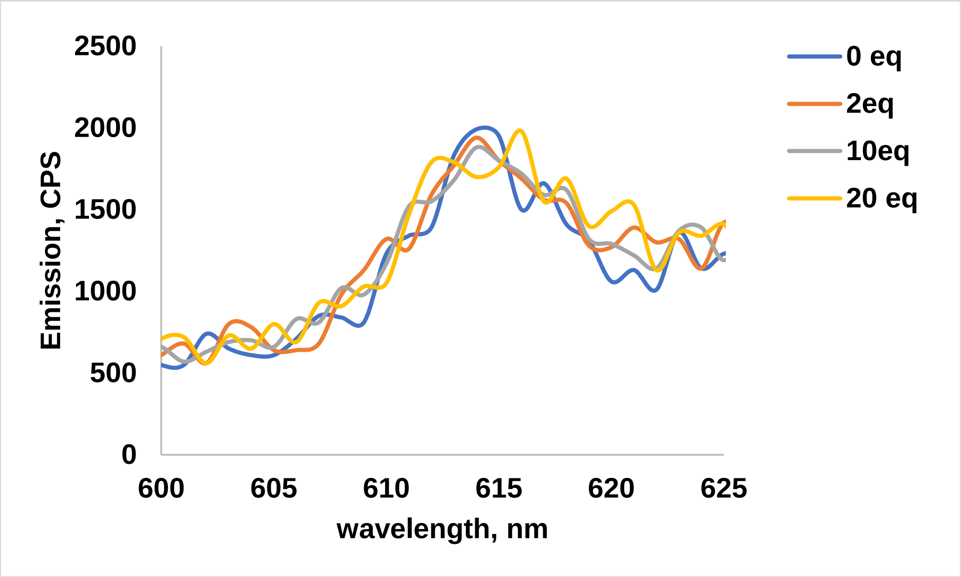

c.

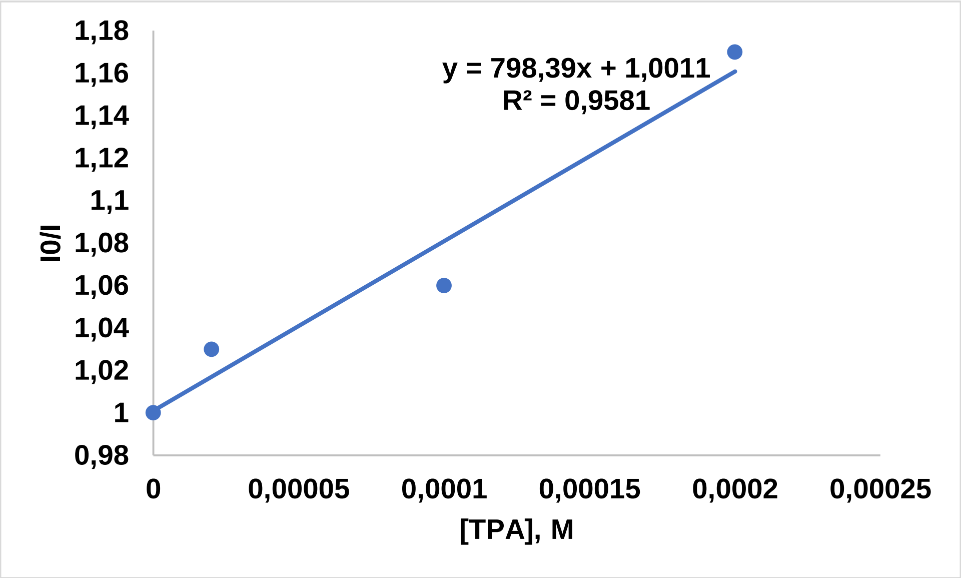

d.

8. Computational Results

8.1. General remarks, geometry optimizations and UV/vis spectra calculations

All visualizations of structures and MOs were created with UCSF Chimera[14] 1.14.0.

Quantum mechanical calculations were performed with the ORCA 4.1.2[15,16] and xtb 6.1.4[17] program packages. Geometries were pre-optimized with the GFN2-xTB[18] extended tight binding method and finally optimized using the PBE0[19] hybrid functional in combination with the triple-ζ def2-TZVP[20] basis set. The numerical quadrature grid m5 grid was employed for the integration of the exchange-correlation contributions and default convergence criteria for energies and gradients were applied as implemented in ORCA. The libint2 library was used for the calculation of two electron integrals.[21]

All geometry optimizations were calculated applying the generalized Born model augmented with the hydrophobic solvent accessible surface area term (GBSA)[22] or conductor-like polarizable continuum solvation model[23] (CPCM) for tetrahydrofurane. All geometry optimizations were performed applying the RIJCOSX[24] approximation (GridX5) for Coulomb integrals[25] with matching default auxiliary basis sets.[26] The D4(EEQ)[27,28] dispersion correction scheme applying Becke-Johnson (BJ) damping[29,30] and including Axilrod-Teller-Muto (ATM)[31,32] type three-body dispersion to the total dispersion energy was applied.

UV/vis spectra were calculated applying time-dependent density functional theory (TD-DFT) (ORCA 4.1.2). For the TD-DFT calculations the PBE0 hybrid functional was applied with the triple-ζ def2-TZVP basis set. The RIJCOSX approximation (GridX5, GridX6 for final energy) was applied to accelerate the TD-DFT calculations. For all molecules the 150 lowest lying states were calculated. All UV/vis spectra were calculated applying CPCM for tetrahydrofurane.

8.2. UV/vis spectra and HOMO-LUMO transitions

Absoption spectra were simulated for the Cp_2TiX_2 complexes (X = F, Cl, Br, OMs, TFA). A shift of 1828.2 cm^{-1} was applied for all complexes to match the energy for the second transition of the calculation to the experimental spectrum of Cp_2TiCl_2.

8.3. Discussion of Anion Effect

Analysis of the computational absorption spectra reveals that the HOMO-LUMO transition of the titanocene species can only be achieved with green light for X = Cl and Br. The other complexes (X = F, OMs, TFA) require shorter wavelengths. As a consequence they are unfit for our green light induced catalysis with titanocenes.

Cp_2TiF_2

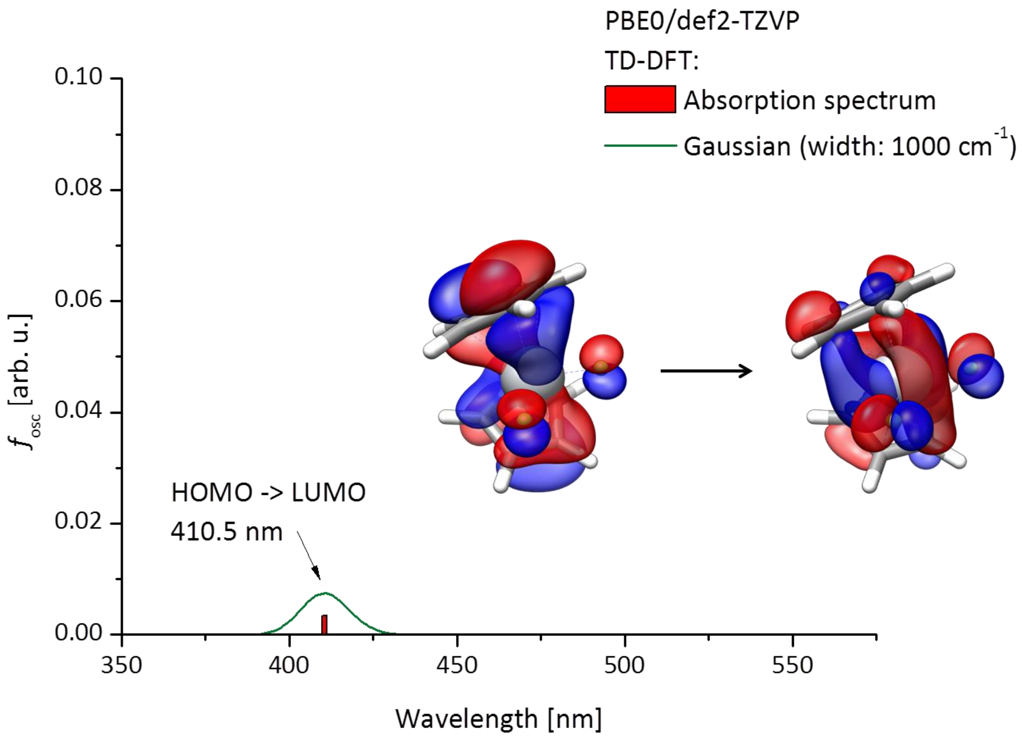

Cp_2TiCl_2

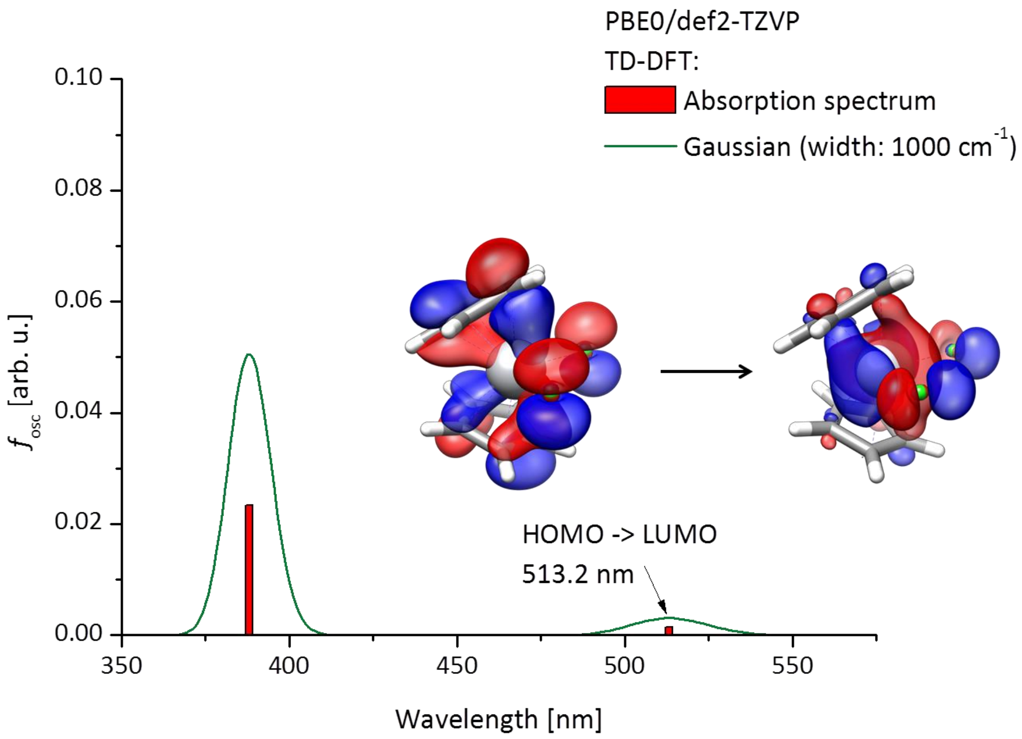

Cp_2TiBr_2

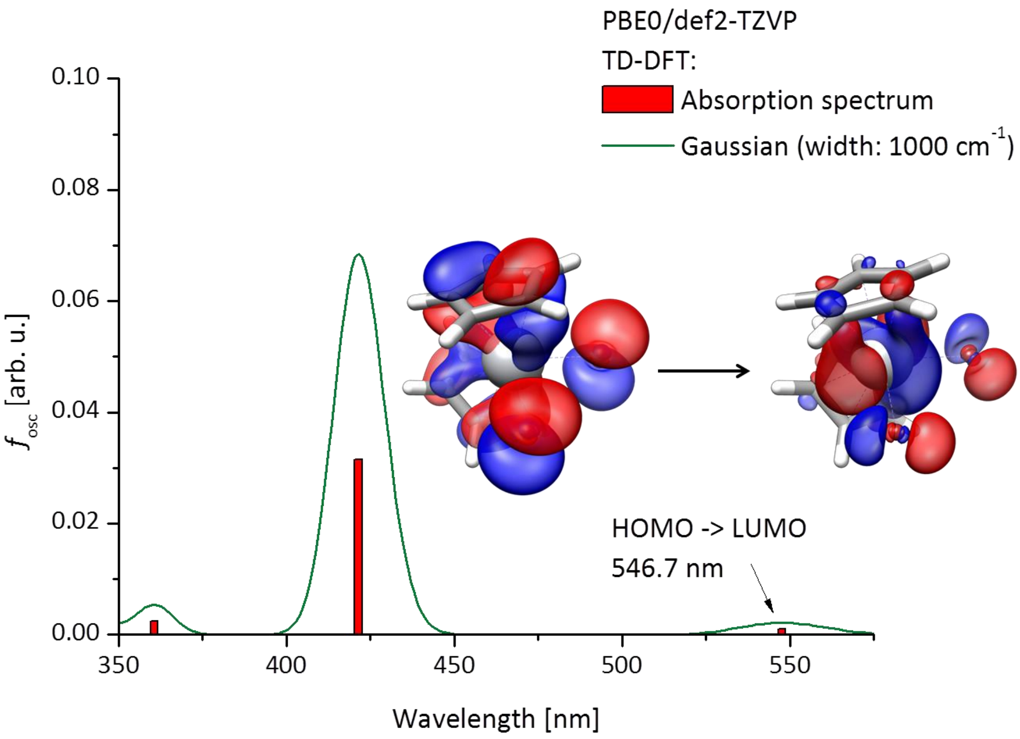

$Cp_2Ti(OMs)_2$

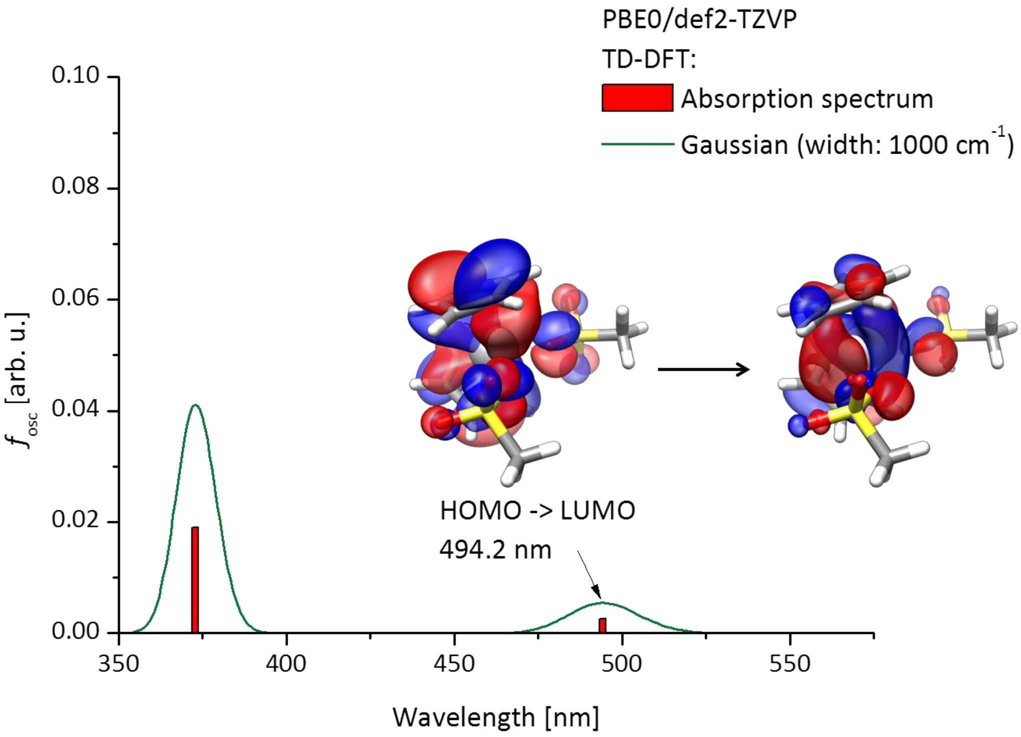

$Cp_2Ti(TFA)_2$

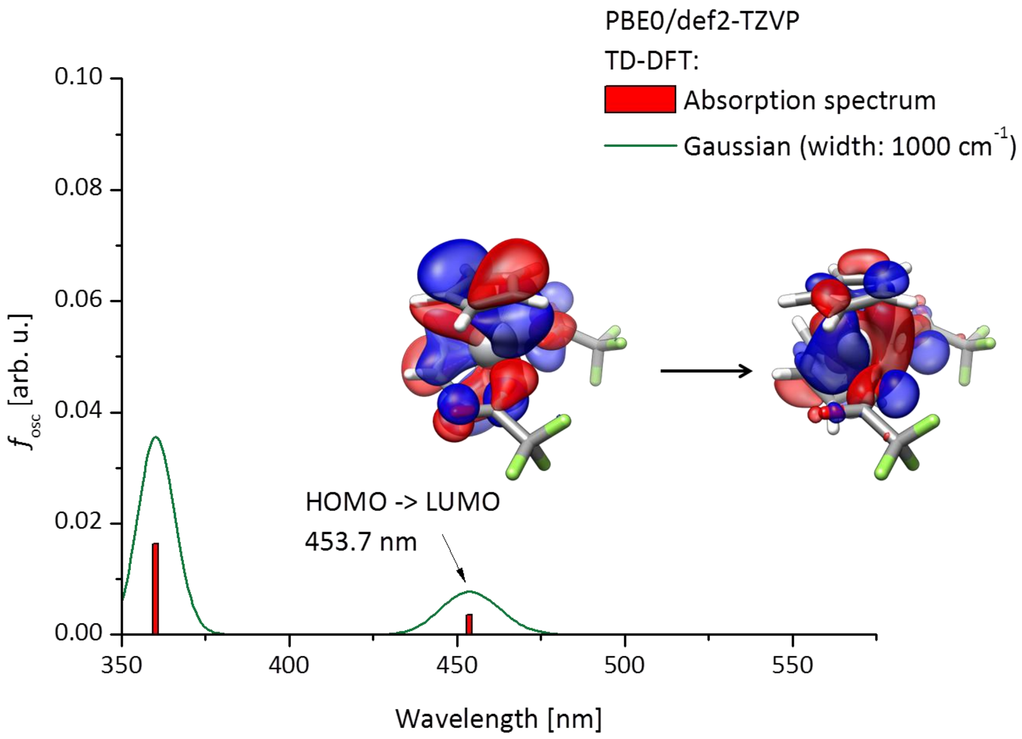

Overview

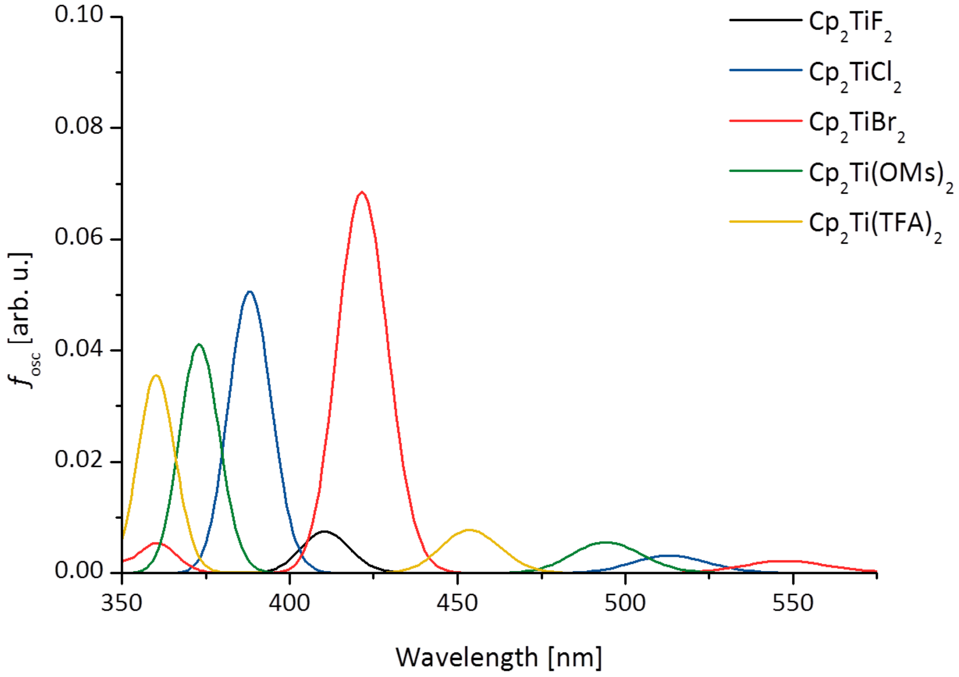

References

[1] A. Gansäuer, H. Bluhm, M. Pierobon, *J. Am. Chem. Soc.* **1998**, *120*, 12849-12859.

[2] T. Sone, A. Yamaguchi, S. Matsunaga, M. Shibasaki, *J. Am. Chem. Soc.* **2008**, *130*, 10078-10079.

[3] A. Gansäuer, A. Barchuk, D. Fielenbach, *Synthesis* **2004**, *2004*, 2567-2573.

[4] R. Davis, A. F. Kluge, M. L. Maddox, M. L. Sparacino, *J. Org. Chem* **1983**, *48*, 255-259.

[5] A. Gansäuer, M. Klatte, G. M. Brändle, J. Friedrich, *Angew. Chem. Int. Ed.* **2012**, *51*, 8891-8894.

[6] Z. Zhang, R. B. Richrath, A. Gansäuer, *ACS Catal.* **2019**, *9*, 3208-3212.

[7] A. Gansäuer, H. Bluhm, T. Lauterbach, *Adv. Synth. Catal.* **2001**, *343*, 785-787.

[8] K. Rajendra Prasad, A. Venkanna, K. S. Babu, A. R. Prasad, J. M. Rao, *Tetrahedron Lett.* **2014**, *55*, 616-618.

[9] A. Gansäuer, M. Pierobon, H. Bluhm, *Synthesis* **2001**, *2001*, 2500-2520.

[10] D. S. G. Henriques, K. Zimmer, S. Klare, A. Meyer, E. Rojo-Wiechel, M. Bauer, R. Sure, S. Grimme, O. Schiemann, R. A. Flowers Ii, A. Gansäuer, *Angew. Chem. Int. Ed.* **2016**, *55*, 7671-7675.

[11] a) T. Toyao, S. M. A. H. Siddiki, Y. Morita, T. Kamachi, A. S. Touchy, W. Onodera, K. Kon, S. Furukawa, H. Ariga, K. Asakura, K. Yoshizawa, K.-i. Shimizu, *Chem. Eur. J.* **2017**, *23*, 14848-14859; b) X. Du, Y. Zhang, D. Peng, Z. Huang, *Angew. Chem. Int. Ed.* **2016**, *55*, 6671-6675.

[12] a) R. Fujihara, K. Nakata, *ChemistrySelect* **2019**, *4*, 75-77; b) K. Kolmakov, E. Hebisch, T. Wolfram, L. A. Nordwig, C. A. Wurm, H. Ta, V. Westphal, V. N. Belov, S. W. Hell, *Chem. Eur. J.* **2015**, *21*, 13344-13356.

[13] W. A. Nugent, T. V. RajanBabu, *J. Am. Chem. Soc.* **1988**, *110*, 8561-8562.

[14] E. F. Pettersen, T. D. Goddard, C. C. Huang, G. S. Couch, D. M. Greenblatt, E. C. Meng, T. E. Ferrin, *J. Comput. Chem.* **2004**, *25*, 1605-1612.

[15] F. Neese, *Wiley Interdiscip. Rev. Comput. Mol. Sci.* **2012**, *2*, 73-78.

[16] F. Neese, ORCA: An Ab Initio, Density Functional and Semiempirical Program Package , V. 4.1.2; MPI Für Chemische Energiekonversion: Mülheim a. d. Ruhr, Germany, **2019**.

[17] S. Grimme, XTB, V. 6.1.4; Mulliken Center for Theoretical Chemistry, University of Bonn. **2019**.

[18] C. Bannwarth, S. Ehlert, S. Grimme, *J. Chem. Theory Comput.* **2019**, *15*, 1652-1671.

[19] C. Adamo, V. Barone, *J. Chem. Phys*. **1999**, *110***,** 6158-6170.

[20] F. Weigend, R. Ahlrichs, *Phys. Chem. Chem. Phys.* **2005**, *7*, 3297-3305.

[21] The library can be accessed from: http://libint.valeyev.net

[22] W. C. Still, A. Tempczyk, R. C. Hawley, T. Hendrickson, *J. Am. Chem. Soc.* **1990**, *112*, 6127-6129.

[23] V. Barone, M. Cossi, *J. Phys. Chem. A* **1998**, *102*, 1995-2001.

[24] F. Neese, F. Wennmohs, A. Hansen, U. Becker, *Chem. Phys.* **2009**, *356*, 98-109.

[25] K. Eichkorn, O. Treutler, H. Öhm, M. Häser, R. Ahlrichs, *Chem. Phys. Lett.* **1995**, *240*, 283-289.
[26] F. Weigend, *Phys. Chem. Chem. Phys.* **2006**, *8*, 1057-1065.
[27] E. Caldeweyher, S. Ehlert, A. Hansen, H. Neugebauer, S. Spicher, C. Bannwarth, S. Grimme, *J. Chem. Phys.* **2019**, *150*, 154122.
[28] E. Caldeweyher, C. Bannwarth, S. Grimme, *J. Chem. Phys.* **2017**, *147*, 034112.
[29] S. Grimme, S. Ehrlich, L. Goerigk, *J. Comput. Chem.* **2011**, *32*, 1456-1465.
[30] A. D. Becke, E. R. Johnson, *J. Chem. Phys.* **2005**, *123*, 154101.
[31] B. M. Axilrod, E. Teller, *J. Chem. Phys.* **1943**, *11*, 299-300.
[32] Y. Muto, *Proc. Phys. Math. Soc. Jpn.* **1943**, *17*, 629-631.
